# The Role of Psychometrics in Individual Differences Research in Cognition: A Case Study of the AX-CPT

**DOI:** 10.3389/fpsyg.2017.01482

**Published:** 2017-09-04

**Authors:** Shelly R. Cooper, Corentin Gonthier, Deanna M. Barch, Todd S. Braver

**Affiliations:** ^1^Cognitive Control and Psychopathology Laboratory, Department of Psychological & Brain Sciences, Washington University in St. Louis, St. Louis MO, United States; ^2^LP3C EA 1285, Department of Psychology, Université Rennes 2 Rennes, France

**Keywords:** psychometrics, variability, reliability, task design, replication, AX-CPT

## Abstract

Investigating individual differences in cognition requires addressing questions not often thought about in standard experimental designs, especially regarding the psychometric properties of the task. Using the AX-CPT cognitive control task as a case study example, we address four concerns that one may encounter when researching the topic of individual differences in cognition. First, we demonstrate the importance of variability in task scores, which in turn directly impacts reliability, particularly when comparing correlations in different populations. Second, we demonstrate the importance of variability and reliability for evaluating potential failures to replicate predicted correlations, even within the same population. Third, we demonstrate how researchers can turn to evaluating psychometric properties as a way of evaluating the feasibility of utilizing the task in new settings (e.g., online administration). Lastly, we show how the examination of psychometric properties can help researchers make informed decisions when designing a study, such as determining the appropriate number of trials for a task.

## Introduction

Creating task paradigms that tap into specific cognitive processes is a formidable challenge. In many cases, when a new cognitive task is developed and is shown to have utility, the task is then administered in a variety of settings and to a variety of populations. Although this is not inherently problematic, researchers need to thoroughly examine whether the ability of a task to effectively measure a construct is maintained or compromised when the task is employed in new situations. In other words, researchers need to ensure that the psychometric properties of the task are preserved. This issue can be rigorously assessed using principles and methods established in the field of psychometrics. Conversely, failure to fully evaluate the psychometric properties of a task can impede researchers from: (a) making optimal study design decisions, (b) finding the predicted results, and (c) correctly interpreting the results they have obtained.

The current study examines four issues that explicitly demonstrate how insufficient understanding of psychometric qualities can hinder researchers interested in individual differences in cognition. The first two issues illustrate how finding different correlations across populations (Issue 1) and across samples (Issue 2) can be misleading when psychometric properties of the task are not considered. The other two issues describe how examination of psychometric characteristics can help researchers decide on a data collection method (Issue 3) and on the appropriate number of trials (Issue 4). The following sections will first highlight relevant principles and methods in psychometric theory, and then describe the cognitive task paradigm used to illustrate these issues—the AX-CPT.

### Psychometric Theory

The measurement qualities of a cognitive task can be summarized with three properties: discriminating power, reliability, and validity. The most basic quality of a test is variability or discriminating power: in other words, its ability to produce a sufficient spread of scores to appropriately discriminate individuals (e.g., Kline, 2015). This property is rarely discussed, perhaps because “there is little need to stress this point, which becomes self-evident if we think of the value of a psychological test on which all subjects scored the same” (Kline, 2015, p. 6). But more subtly, a test demonstrating a restricted range of scores (for example, a ceiling or floor effect) can also be said to lack discriminating power, meaning that it will have low sensitivity for detecting individual differences. Variability is often assessed as the range or variance of observed scores on the test.

Reliability is defined in the context of Classical Test Theory (CTT), which states that the observed variance in a measurement (X) is the sum of true score variance (T) attributable to psychological characteristics of the participant, and random measurement error (E). This idea is usually summarized as (*X* = *T* + *E*). The reliability of a measurement is defined as the proportion of true variance: in other words, the ratio of true score variance to total observed score variance $\left(r_{xx}=\frac{\sigma_{T}^{2}}{\sigma_{X}^{2}}\right)$. In short, reliability indicates to what extent the scores produced by the test are subject to measurement error. Reliability can be estimated with four methods (internal consistency, test–retest, parallel forms, and inter-rater reliability). Two of these methods are particularly relevant here. Internal consistency refers to the extent to which items or trials within an instrument all yield similar scores; this is estimated based on indices such as Cronbach’s alpha (α). As the name implies, the test–retest method evaluates the stability of scores obtained over multiple administrations of the same instrument to the same individuals.

Lastly, a test is said to be valid when it actually measures what it purports to measure. Establishing validity of a test is an extensive process, which requires researchers to ensure—among other things—that the nature and content of the test appropriately reflects the construct it is supposed to assess, and that the test demonstrates the expected relationships with other measures. Ultimately, the essential purpose of a test is to be valid. Critically for our purposes, however, the three basic psychometric properties are organized hierarchically. Validity is contingent upon reliability: a test that is contaminated by measurement error to a large extent cannot accurately measure what it is supposed to measure. Likewise, reliability is contingent upon discriminating power. By definition, a reliable measurement tool is one in which there is a large amount of true score variance. If the test yields scores with little to no variability, then there can be little to no true score variance. All other things being equal, the reliability of a measure decreases when the variance of observed scores decreases (e.g., Cronbach, 1949). This phenomenon is akin to the effect of restriction of range on correlations.

Another critical point is that psychometric properties characterize the scores produced by a test in a particular setting, not the test itself, and though this point has been frequently reiterated in the psychometric literature (Feldt and Brennan, 1989; Wilkinson and Task Force on Statistical Inference, 1999; Caruso, 2000; Yin and Fan, 2000), it bears repeating. In other words, the same test may demonstrate different psychometric properties altogether in different contexts. For example, a test may be too easy for participants in one population, leading to low discriminating power, unreliable scores, and ultimately low validity. On the other hand, the same test may demonstrate excellent validity in a different population of participants with lower ability levels. As a consequence, researchers need to explore the psychometric properties of a task in each of the different populations they intend to compare: a task that is optimized for individual differences analyses in one group may not have the same utility for a different population.

Many studies fail to examine or report reliability estimates, especially studies interested in experimental manipulations. Part of the reason may be that the existence of group effects is taken to imply that the task functions as intended. However, demonstrating experimental variation in a measure only suggests that scores are not entirely random; this does not mean that the scores are precise estimates of a participant’s ability. Thus, large between-group effect sizes do not imply that a task is reliable and do not provide sufficient information regarding the quality of the measure for individual differences research.

Those studies that do report reliability typically do not scrutinize variability of the scores: observed variance is usually considered as a source of noise, or as an error term. However, both properties are important and can affect interpretation of the results. A test with low discriminating power in a given sample has little value from the perspective of individual differences research. Estimating variability is also important to contextualize reliability estimates, since low discriminating power reduces the reliability of the measure; this is all the more important given that discriminating power can vary across samples. Reliability, as a reflection of measurement error, directly influences the effects that can be observed in a given experiment. While this holds true for experimental manipulations, it is perhaps even more critical for individual differences studies. Experimental designs are usually interested in group averages: in this case, measurement error inflates random variance (or in other words, reduces statistical power to observe effects of interest), a problem that can be canceled out by increasing sample size. On the other hand, individual differences studies are interested in the precise score of each individual, which means that obtaining accurate individual measurements is more of a concern: for example, correlations between a test and other measures decrease as a function of the square root of reliability (e.g., Nunnally, 1978). In the current study, we examine issues of variability and reliability within the context of the AX-CPT task, which is described next.

### Cognitive Control and the AX-CPT

The AX-CPT is a variant of the continuous performance task (CPT; Servan-Schreiber et al., 1996), and is commonly used in cognitive control experiments (Barch et al., 2009; Carter et al., 2012). Cognitive control is thought to be a critical component of human high-level cognition, and refers to the ability to actively maintain and use goal-directed information to regulate behavior in a task. Cognitive control is thus used to direct attention, prepare actions, and inhibit inappropriate response tendencies. Importantly for the current paper the domain of cognitive control is thought to be one in which individual differences make a large contribution to observed performance (Miyake et al., 2000; Kane and Engle, 2002; Burgess et al., 2011; Salthouse, 2016).

The AX-CPT has been used in many studies and has played an important role in the development of a specific theoretical framework, known as the Dual Mechanisms of Control (DMC; Braver et al., 2007; Braver, 2012). The DMC framework proposes that there are two ways to implement cognitive control: proactive, where control is implemented in advance through active maintenance of contextual information, and reactive, where control is implemented after an event has occurred. One of the main assumptions of the DMC framework is that there are likely stable individual differences in the proclivity to use proactive or reactive control (Braver, 2012). For example, non-clinical young adults tend to preferentially use proactive control (Braver, 2012). Moreover, the ability and/or preference to use proactive control is likely to be influenced by other cognitive abilities that index how easily and flexibly one can maintain context information. For instance, a participant with below average working memory capacity (WMC) could have trouble actively maintaining context cues, and thus be biased toward using reactive control strategies; whereas a participant with above average WMC may not find maintaining contextual information particularly taxing, and therefore may lean toward using proactive control strategies. Prior studies have reported relationships between performance on the AX-CPT (and similar tasks) and individual differences in WMC (Redick, 2014; Richmond et al., 2015), fluid intelligence (Gray et al., 2003), and even reward processing (Jimura et al., 2010).

The AX-CPT is designed to measure cognitive control in terms of how context cues are actively maintained and utilized to direct responding to subsequent probe items. Participants are instructed to make a certain response for a target probe, and a different response for all non-target probes. The target probe is the letter *X*, but only if it was preceded by the letter *A* as the context cue. This naturally leads to four trial types: AX (target), AY, BX, and BY, where “B” represents any letter other than *A* and “Y” represents any letter other than *X*. The classic AX-CPT paradigm includes 70% of AX trials, and 10% each of AY, BX, and BY trials (Braver et al., 2007). More recent versions of the task have used different proportions of trials (Richmond et al., 2015; Gonthier et al., 2016), but the higher proportion of AX trials relative to AY and BX trials is always maintained. This creates a prepotent tendency to make a target response following both A cues and X probes.

Researchers use the AX-CPT to explore differences in proactive vs. reactive control by examining AY and BX trials. In participants utilizing proactive control, the context provided by the cue is particularly helpful for correctly responding to BX trials, since the cue fully determines that the trial will be non-target. Yet a proactive strategy also leads to more AY errors because participants often incorrectly prepare for a target probe in the presence of an A-cue. By contrast, AY trials are less difficult and BX trials are more difficult for participants using reactive control, as they do not actively prepare a response during the interval between the cue and the probe.

### Psychometrics and the AX-CPT

From a psychometric standpoint, the AX-CPT demonstrates two special features. First, its very design makes certain types of trials rarer than others: in the classic version of the task, AX trials are seven times more frequent than other trial types. The low number of trials for AY and BX trials poses a special challenge to precise estimation of performance. This is especially important because the two least frequent trial types are also the two most critical to disentangling proactive and reactive control. Second, young adults tend to rely mainly on proactive control, which yields very high performance on all trial types but AY. In other words, the task tends to elicit ceiling effects on certain trial types, resulting in low discriminating power (Gonthier et al., 2016). Due to these features, the AX-CPT and its variants are particularly prone to demonstrating poor psychometric properties in healthy young adults, especially for indices based on accuracy. While prior studies have found reliabilities around or above 0.70 on AY and BX trials in schizophrenia cohorts (Henderson et al., 2011; Strauss et al., 2014), reliabilities below 0.60 have been reported for AY and BX trials in healthy young adults (Rush et al., 2006; Henderson et al., 2011). Thus, the AX-CPT is an interesting candidate task for a case study of the importance of considering psychometric properties in cognitive research related to individual differences. The goal of the current study is to demonstrate how careful examination of psychometric characteristics can impact the interpretation of individual differences results and aid researchers in making optimal study design decisions. We examine four different issues that researchers may encounter.

To examine these issues, we use AX-CPT datasets collected in different samples and in different labs with different versions of the task, and we systematically assess variability and reliability of the measures. Variability is indexed as the observed variance of the scores; reliability is assessed with the internal consistency method, as well as the test–retest method when available. Performance on the AX-CPT can be measured based on accuracy or response times (RTs); for simplicity, we restrict our study to accuracy. Psychometric analyses of RTs are not included here, even though they are often used as cognitive control indices in the AX-CPT, for three reasons. First, there is more variability in RTs than accuracy rates for limited number of trials, and RTs typically come with less of a ceiling effect; as result, RTs tend to demonstrate higher reliability than accuracy rates and would make for a more limited case study. Second, RTs are typically only computed for correct response trials, complicating the computation of internal consistency indices (since different individuals have different numbers of trials). Third, observed RTs present more of a measurement challenge, since they reflect not only the cognitive demands of a given condition or trial type, but also serve as a general index of processing speed, which is a highly stable and robust individual difference component. Typically, this issue is addressed through difference scores (i.e., subtracting a low demand condition from the high demand), but then this presents new challenges for estimating reliability (Rogosa and Willett, 1983). Thus, calculating the reliability of RT indices could produce either higher or lower estimates than accuracy indices for potentially artifactual reasons. Because such issues are beyond the scope of the current paper, we do not address them in the main text. However, for archival purposes we include Supplemental Materials that provide reliability estimates of raw RTs as well as common derived measures in both RT and accuracy, including the signal detection index *d*′-context and the proactive behavioral index.

## Issue 1: Psychometric Properties of A Measure Can Complicate Between-Populations Findings

One of the “gold standard” experimental designs is to compare the performance of two different groups on the same task. As such, it follows that one might also want to examine individual difference relationships between the task and some outcome measure of interest, comparing such relationships across the two groups. The study detailed in this section was interested in the relationship between individual differences in AX-CPT performance and episodic memory function, using an encoding and retrieval task. Two different groups were compared: a schizophrenia cohort and a matched control group. Therefore, Issue 1 examines variability and reliability (test–retest reliability and internal consistency reliability) of the AX-CPT, when administered to both participants with schizophrenia and matched controls. The comparison highlights a key issue: evaluation of a task and its ability to provide information regarding relative individual difference relationships between groups requires an understanding of the variability and reliability present in each group. That is, assuming that the same exact task can be used to examine individual differences in two different populations may lead to erroneous inferences, since the psychometric characteristics of the task may vary across populations.

### Methods

#### AX-CPT Datasets

As part of the Cognitive Neuroscience Test Reliability and Clinical applications for Schizophrenia (CNTRaCS) consortium (Gold et al., 2012), Strauss et al. (2014) published a study whose stated goal was to explore the temporal stability, age effects, and sex effects of various cognitive paradigms including the AX-CPT. A cohort of 99 schizophrenia participants and 131 controls matched on age, sex, and race/ethnicity were administered the CNTRaCS tasks across three sessions, with both groups completing identical versions of the task. The CNTRaCS battery included several other tasks, including the Relational and Item-Specific Encoding (RISE) task (Ragland et al., 2012). We chose to use the RISE, since it has been used to index strategic aspects of episodic memory that may have construct similarity to cognitive control. In particular, prior research has indicated some shared variance across the AX-CPT and the RISE, which was interpreted as reflecting common demands for prefrontally mediated cognitive control (Gold et al., 2012). There are three primary RISE conditions considered here: associative recognition, item recognition associative encoding, and item recognition item encoding. The same versions of the RISE and AX-CPT were administered to both cohorts. The design of this variant of the AX-CPT elicited particularly prepotent target responses, with 104 AX trials, 16 AY trials, 16 BX trials, and 8 BY trials (144 total trials).

#### Analyses

The first set of analyses aimed to understand the relationship between the AX-CPT and the RISE, as a function of population. We first correlated AX-CPT accuracy for all trial types with RISE accuracy for all conditions, after averaging performance across the three time points (e.g., correlation of the average of BX accuracy and the average of IRAE accuracy), separately for the two groups of participants. This analysis comprised the 89 schizophrenia patients and 117 controls that completed the AX-CPT and the RISE at all three time points. Fisher tests were used to determine whether correlations were significantly different between the control and schizophrenia cohorts.

The second set of analyses examined the psychometric characteristics of AX-CPT measures for each group, using 92 schizophrenia and 119 control participants that completed all three time points of the AX-CPT. Discriminating power was indexed with observed variances of the scores for each trial type. Differences in observed variances between schizophrenia and control cohorts were examined via Brown–Forsythe tests (Brown and Forsythe, 1974). Internal consistency reliability was assessed with Cronbach’s α for each trial type at each time point. Bootstrapped 95% confidence intervals based on 1000 bootstrapped resamples were computed using the *ltm* package in R (Rizopoulos, 2006). In order to fully exploit the multi-wave structure of this dataset, we placed emphasis on test–retest reliability, which was estimated with the intraclass correlation coefficient (ICC; ICC2k), including 95% confidence intervals. A significant difference in ICCs was defined as non-overlapping 95% confidence intervals. The same procedures were used to evaluate test–retest reliability ICCs for the RISE.

Lastly, we calculated the upper bound correlations that could have possibly been obtained between the AX-CPT and the RISE using the following formula: $r_{UB}=1*\sqrt{r_{xx}\cdot r_{yy}}$ (Spearman, 1904), where *r*_UB_ is the upper bound correlation between *x* and *y*, and *r*_xx_ and *r*_yy_ are the reliability coefficients for *x* and *y*, respectively.

### Results

We first investigated the correlation between the AX-CPT and the RISE, and found that every single correlation coefficient was larger in the schizophrenia cohort than the control cohort (**Table 1**). We then tested whether correlation coefficients were significantly larger in the schizophrenia cohort than controls (one-tailed). **Table 1** shows the correlation coefficients for each AX-CPT trial type and each RISE condition. Four out of 12 possible comparisons were significant, with four others trending toward significance (i.e., *p*-values of 0.10 or less; **Table 1**).

**Table 1:** Correlations between the AX-CPT and the RISE as a function of population.

RISE condition	AX-CPT trial type	*r* CTRL	*r* SCZ	*z*	*P*	*r*_UB_ CTRL	*r*_UB_ SCZ
Associative recognition	AX	0.18	0.25	-0.47	0.318	0.76	0.85
	AY	0.00	0.21	-1.46	0.072^+^	0.71	0.84
	BX	0.25	0.26	-0.04	0.486	0.76	0.79
	BY	0.06	0.23	-1.17	0.121	0.72	0.80
Item recognition Associative encoding	AX	0.27	0.41	-1.06	0.144	0.76	0.85
	AY	0.15	0.32	-1.26	0.103^+^	0.71	0.85
	BX	0.18	0.40	-1.70	0.044^∗^	0.76	0.80
	BY	0.09	0.37	-2.12	0.017^∗^	0.72	0.80
Item recognition Item encoding	AX	0.30	0.49	-1.54	0.062^+^	0.78	0.87
	AY	0.13	0.33	-1.51	0.066^+^	0.73	0.86
	BX	0.17	0.44	-2.08	0.019^∗^	0.78	0.81
	BY	0.07	0.39	-2.39	0.008^∗^	0.74	0.82


*Asterisks (∗) indicate significance at *p* < 0.05, crosses (+) indicate trends toward significance, *r* represents correlation coefficient, and *r*_UB_ represents the upper bound correlations. All results here were based on subjects who completed all three time points for both the AX-CPT and the RISE (*n* CTRL = 117, *n* SCZ = 89).*

**FIGURE 1:**
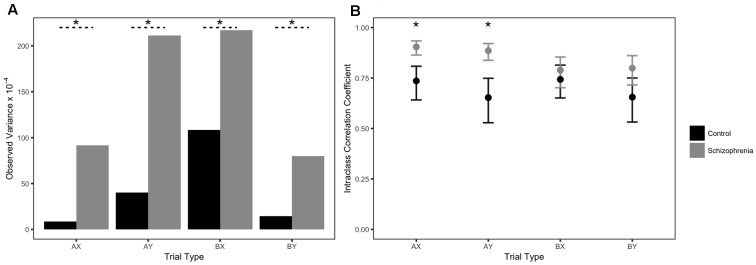
Observed variances and test–retest reliability estimates of AX-CPT accuracy: Issue 1. Error bars represent 95% confidence intervals. Asterisks (^∗^) indicate significant Brown–Forsythe tests at *p* < 0.05, or non-overlapping 95% confidence intervals.

**Table 2** contains descriptive statistics for each cohort (across the three sessions). Please see Supplementary Table S1a for skew and kurtosis values. Controls had higher mean accuracies and smaller standard deviations for all trial types compared to the schizophrenia group. Brown–Forsythe tests for observed variances confirmed that the schizophrenia cohort had significantly larger variances for all trial types compared to controls [AX *F*(91,118) = 21.80, *p* < 0.001; AY *F*(91,118) = 21.91, *p* < 0.001; BX *F*(91,118) = 6.15, *p* = 0.014; and BY *F*(91,118) = 10.88, *p* = 0.001; **Figure 1A**).

**Table 2:** Descriptive statistics of AX-CPT accuracy: Issue 1.

Group	N subjects (% female)	Mean age (range)	Trial type	Mean accuracy	Variance × 10^-4^	Min	Max
CTRL	119 (49.6%)	38.88 (18–65)	AX	0.97	8.42	0.86	1.00
			AY	0.94	40.11	0.69	1.00
			BX	0.91	108.35	0.40	1.00
			BY	0.98	14.42	0.75	1.00
SCZ	92 (41.3%)	39.79 (18–59)	AX	0.92	91.78	0.41	1.00
			AY	0.88	211.16	0.33	1.00
			BX	0.84	217.33	0.27	1.00
			BY	0.95	79.77	0.29	1.00


*For simplicity, descriptive statistics were computed on accuracy averaged over the three sessions.*

Reliability estimates for the AX-CPT are reported in **Table 3**. Out of all the reliability estimates—internal consistency alphas at each time point and test–retest ICCs—there was only one instance of controls showing better reliability than the schizophrenia group (α for BX trials at T1; **Table 3**). The schizophrenia group exhibited higher reliability estimates in all other cases (**Table 3**). **Figure 1B** highlights this reliability difference by visually illustrating the test–retest ICC effects. Non-overlapping 95% confidence intervals on AX and AY trials indicated that ICCs were significantly higher in the schizophrenia group than the control group. Test–retest reliabilities of the RISE were numerically higher for the schizophrenia group than for controls, though not significant based on overlapping 95% confidence intervals. The following pairs contain ICCs for controls and schizophrenia, respectively, for each of the three RISE conditions: associative recognition -0.78, 0.80; item recognition associative encoding -0.78, 0.81; and item recognition item encoding -0.82, 0.84.

**Table 3:** Internal consistency and test–retest reliability estimates: Issue 1.

Group	Trial type	T1 α	T2 α	T3 α	T1–T2–T3 ICC
CTRL	AX	0.82	0.78	0.81	**0.74**
	AY	0.43	0.47	0.68	**0.65**
	BX	0.79	0.65	0.73	**0.74**
	BY	0.14	0.21	0.32	**0.66**
SCZ	AX	0.95	0.93	0.95	**0.90**
	AY	0.77	0.80	0.80	**0.89**
	BX	0.72	0.81	0.81	**0.79**
	BY	0.50	0.55	0.67	**0.80**


Upper bound correlations between the AX-CPT and RISE measures can be found in **Table 1** so readers can easily compare the upper bound vs. observed values. As before, all upper bound correlations are larger in the schizophrenia group than the control group.

### Discussion

Based on the data reported by Strauss et al. (2014), a reasonable conclusion would be that the nature of the relationship between AX-CPT performance and RISE performance is fundamentally different for the schizophrenia cohort than for the control cohort, as suggested by the larger observed correlations (as shown in **Table 1**). This inference is potentially erroneous, however, and highlights the necessity for examining psychometric characteristics like variability and reliability within each population. Here, it is not valid to draw the conclusion that the individual differences relationships are fundamentally different between the two groups, because the reliability of the AX-CPT was significantly lower in the control group for AX and AY trials, and numerically lower for BX and BY trials (**Table 3** and **Figure 1A**). Since low reliability reduces the magnitude of correlations, it is unclear whether the relationship between the AX-CPT and the RISE is actually different for the different populations, or whether the differential correlations are an artifact of low reliability in the control group. In short, simply because a task is appropriate for the measurement of individual differences in one population does not mean that it is good for a different population—the psychometric properties of a task need to be constrained to the population under study.

The differences in reliability may be traced back to differences in variability of the scores. Here, the control group had a much narrower range of scores and exhibited ceiling effects, unlike the schizophrenia group. There was more between-subject variability in the schizophrenia sample than the control sample, which in turn allowed for more variance to be potentially shared between trials (internal consistency reliability) and/or sessions (test–retest reliability). Thus, the larger variability of scores in the schizophrenia group directly contributed to the higher reliability estimates, and ultimately the increase in correlations between the AX-CPT and the RISE. Ceiling-level accuracy rates may be desirable if interrogating RT, since more correct trials would maximize the number of trials that can be used in RT analyses; when using accuracy rates to index individual differences, however, such a ceiling effect directly detracts from the usefulness of the task.

A study by Henderson et al. (2011) gives another example of how an AX-CPT-like task can have differing psychometric characteristics between control and schizophrenia populations. They examined the test–retest reliability of individual trial types for different versions of the Dot Pattern Expectancy task, which is a variant of the AX-CPT in which stimuli are composed of Braille dots rather than letters (MacDonald et al., 2005). The various task versions differed in their inter-stimulus interval (ISI) and in their proportion of trial types. In the version of the task that they concluded was most optimal (Short form #1), they too found that reliability estimates were higher for schizophrenia participants than for matched controls on all trial types (AX—0.90 vs. 0.80, AY—0.65 vs. 0.39, BX—0.79 vs. 0.53, and BY—0.28 vs. 0.21, respectively for patients and controls; see Table 2 in Henderson et al., 2011). In this study too, higher reliability estimates appeared in the context of lower accuracy and much higher variances for schizophrenia patients. While Henderson et al. (2011) accomplished their goal in finding a version that works well for schizophrenia patients, their best version fell short for controls. If one wanted to use their preferred variant for investigating differential correlations between schizophrenia and control populations, that study would likely suffer from the same issues described here—namely, that different psychometric characteristics across populations interferes with interpreting differential correlations.

## Issue 2: Psychometric Characteristics of A Task Can Impact Replication Attempts

As described above, the psychometric properties of a task can complicate the interpretation of between-populations individual differences. Importantly, this is also true for samples taken from the same population. This is especially problematic for situations in which hypothesized relationships fail to materialize or replicate. While the recent “replication crisis” in Psychology has mainly focused on issues such as *p*-hacking, the file drawer problem, insufficient power, and small sample sizes (Open Science Collaboration, 2015), the minimal attention given to the psychometric properties of studied tasks may also be a contributing factor: a measure with low reliability is largely contaminated with error variance, which can lead to decreased effect sizes, as illustrated above. Issue 2 demonstrates how careful inspection of a task paradigm’s psychometric qualities can be useful to interpret within-population replication failures. Here we illustrate this point in terms of the relationships between individual differences in performance on the AX-CPT and WMC in two different datasets.

### Methods

#### AX-CPT Datasets

Richmond et al. (2015) hypothesized that participants with high WMC, as measured by operation span and symmetry span (Unsworth et al., 2005; Redick et al., 2012), would be more inclined to engage in proactive control strategies. The first dataset we used in the current study is the data from Richmond et al.’s (2015) Experiment 1, collected at Temple University. Going forward, we refer to this as the “Temple” dataset. This data consists of 104 participants ranging from 18 to 26 years old, with the sample coming from the local student community. This AX-CPT variant comprised 58 AX, 14 AY, 14 BX, and 58 BY trials (144 total trials).

Experiment 2 of Gonthier et al. (2016) also measured WMC with operation span and symmetry span tasks, and included a standard condition of the AX-CPT very similar to the Temple study. The second dataset we used in the present study is this standard condition of the AX-CPT, which we will refer to as the “Savoy” dataset, as these data were collected at the University of Savoy in France. The trial type proportions in the AX-CPT task used in the Savoy study were the same as the Temple dataset, but the overall number of trials was slightly lower in the Savoy study: 40 AX, 10 AY, 10 BX, and 40 BY (100 total trials). The Savoy study included 93 native French speakers ranging from 17 to 25 years old.

#### Analyses

The first set of analyses examined correlations between AX-CPT and WMC for the two samples. As in Issue 1, Brown–Forsythe tests were then conducted to determine if there were any significant differences in observed variances between the two datasets. Internal consistency reliability was estimated using Cronbach’s α with the same procedure outlined in Issue 1. Using methods from Feldt et al. (1987), chi-square tests were conducted using the *cocron* package in R (Diedenhofen and Musch, 2016) in order to determine whether α estimates from the two samples were statistically different from each other.

### Results

In the Temple dataset, WMC significantly correlated with higher accuracy on AX, BX, and BY trials [AX *r*(102) = 0.36, *p* < 0.001, BX *r*(102) = 0.39, *p* < 0.001, and BY *r*(102) = 0.28, *p* = 0.004]. Although the Savoy dataset was very similar to the Temple dataset with nearly identical task structure, BX accuracy was the only trial type in the Savoy study to significantly correlate with WMC [AX *r*(91) = 0.05, *p* = 0.628, BX *r*(91) = 0.38, *p* < 0.001, and BY *r*(91) = -0.03, *p* = 0.799]. Thus, the Savoy experiment did not obtain the same results as the Temple experiment for AX and BY trials. Fisher tests confirmed that correlations with WMC were significantly larger in the Temple study than in the Savoy study for both AX and BY trial types (AX *z* = -2.26, *p* = 0.012 and BY *z* = -2.17, *p* = 0.015).

**FIGURE 2:**
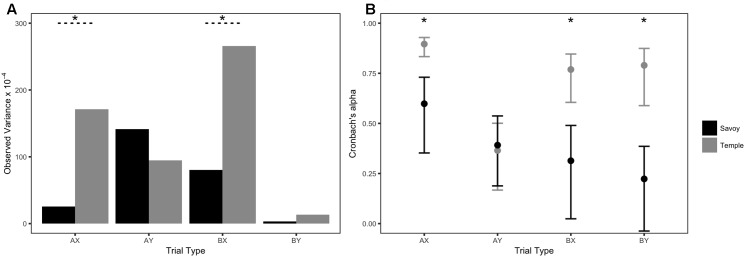
Observed variances and internal consistency reliability estimates of AX-CPT accuracy: Issue 2. Error bars represent 95% confidence intervals. Asterisks (^∗^) indicate significant Brown–Forsythe tests or significant Feldt tests at *p* < 0.05.

Descriptive statistics are reported in **Table 4**. Please see Supplementary Table S1b for skew and kurtosis values. Participants in the Savoy dataset performed remarkably well on all AX-CPT measures, with no trial types falling below a mean accuracy of 90%. The range of mean accuracies was larger in the Temple data than the Savoy data for all trial types, and all trial types except AX had larger standard deviations (**Table 4**). Brown–Forsythe tests of observed variances (**Figure 2A**) were significant for AX and BX trial types [AX *F*(103,92) = 23.66, *p* < 0.001 and BX *F*(103,92) = 6.05, *p* = 0.015], in the direction of Temple having larger variances than Savoy. The observed variance of BY trials was also larger in the Temple study, trending toward significance [*F*(103,92) = 3.36, *p* = 0.068]. Observed variance of AY accuracy was larger in the Savoy study than the Temple study, though the difference was non-significant [*F*(92,103) = 1.59, *p* = 0.208].

**Table 4:** Descriptive statistics of AX-CPT accuracy and internal consistency reliability estimates: Issue 2.

Group	N subjects (% female)	Mean age (range)	Trial type	Mean accuracy	Variance × 10^-4^	Min	Max	α
Savoy	93 (78.5%)	20.18 (17–25)	AX	0.96	25.43	0.72	1.00	0.60
			AY	0.90	141.26	0.50	1.00	0.39
			BX	0.94	80.50	0.60	1.00	0.31
			BY	0.99	3.14	0.93	1.00	0.22
Temple	104 (73.4%)	21.33 (18–25)	AX	0.86	171.01	0.38	1.00	0.90
			AY	0.90	94.53	0.57	1.00	0.36
			BX	0.88	265.96	0.21	1.00	0.77
			BY	0.98	13.27	0.74	1.00	0.79


As seen in **Figure 2B**, internal consistency reliability estimates of AX-CPT accuracy were statistically different for AX trials [χ^2^(1) = 3.85, *p* < 0.001], BX trials [χ^2^(1) = 2.97, *p* < 0.001], and BY trials [χ^2^(1) = 3.69, *p* < 0.001], in the direction of Savoy having lower alphas than Temple. Alphas were not significantly different for AY trials [χ^2^(1) = 1.04, *p* = 0.830].

### Discussion

The Savoy study found some, but not all aspects of relationships between AX-CPT and WMC reported by the Temple study. In the Temple dataset, AX, BX, and BY trials were found to significantly correlate with WMC. In the Savoy dataset, the only trial type that correlated with WMC was BX, even though this dataset had a similar sample size, procedure, and was also performed in a similar population of young adults (mostly college students). Yet after careful consideration of the psychometric properties of the task within each sample, it is not necessarily surprising that correlations with AX and BY trials failed to replicate; reliability was also significantly lower for these two trial types in the Savoy study. As discussed in Issue 1, reliability elicits larger effect sizes, which is critical for individual differences designs. Lower reliability coefficients in the Savoy study indicated that even if there were a real relationship between AX-CPT accuracy and WMC on AX and BY trials, the Savoy study would have had lower sensitivity to detect such a relationship. Despite these datasets being from (at least seemingly) the same population, the reliability of the AX-CPT task varied enough across samples to impede a replication attempt.

As in Issue 1, the differences in reliability may be traced back to differences in the variability of the scores. One characteristic of the Savoy data is that accuracy was near ceiling levels, and as such had very little observed variance, whereas the Temple data had lower mean accuracies and more observed variance (**Table 4**). This reinforces the need to take variability into consideration along with reliability. For example, consider here that internal consistency reliability of AX accuracy in the Savoy dataset was α = 0.60, below the usual threshold of 0.70. A researcher obtaining an α = 0.60 might do one of two things: (1) choose not to use the data from this task for future analyses; or (2) choose to proceed with conducting statistical analyses using these data, and simply report the reliability estimate in the manuscript. In the first scenario, the researcher would likely want to further explore why the reliability estimate was so low to begin with. In the second scenario, the researcher might have trouble interpreting results of the analyses. In both cases, low reliability estimates should motivate researchers into a more careful consideration of other psychometric characteristics, and we recommend exploring variability as a starting point.

It is interesting to note that accuracy on BX trials was significantly correlated to WMC for both datasets, despite the Savoy dataset demonstrating worse psychometric characteristics than the Temple dataset. This finding emphasizes that just because psychometric characteristics are poorer does not necessarily mean that a correlation will not be found. Rather, having poorer psychometric characteristics indicate that there is a decreased probability of detecting a correlation. This is analogous to experimental power. Reduced power simply means that there is a lower probability of detecting a true effect; it does not mean that a true effect cannot be detected.

Here we highlighted that probing the psychometric characteristics of task may lead to changes in the interpretation of correlational findings, which are especially important in replication designs. This is true when comparing correlations across populations (Issue 1), and across samples within the same population (Issue 2). We recommend that future studies report variability and reliability estimates of the tasks employed, especially for studies taking an individual differences approach. Doing so will be especially useful for others trying to replicate hypothesized correlations and to delve deeper into individual differences.

## Issue 3: Psychometric Characteristics Can Help Decide on A Data Collection Method

Many cognitive psychologists are exploring new fast and cost-effective ways to collect behavioral data, including online platforms such as Amazon’s Mechanical Turk (MTurk). Using MTurk as an example, participants from across the world can log in to their Amazon MTurk account, identify “human intelligence tasks” (or HITs), complete the tasks at home, and receive payment seamlessly. These platforms are especially appealing to researchers interested in individual differences questions, since they vastly expand the potential subject pool. However, the utility of online cognitive tasks for individual differences applications is still relatively unexplored. For instance, it is unclear whether reliability of the measures could be compromised due to technical issues surrounding experiments performed on the Internet, and more generally out of the lab. Crump et al. (2013) investigated the validity of using MTurk for conducting cognitive experiments, and showed successful replication of some of the classic cognitive findings including the Stroop, switching, flanker, Simon, and Posner cuing tasks (Crump et al., 2013). However, psychometric properties of the tasks were not reported. Importantly, if researchers are considering adapting a cognitive task paradigm to a new testing environment, one major way to decide whether the task is appropriate for use in the new setting is by comparing its psychometric characteristics in each context. If there are no differences in the variability and reliability of the task administered in different contexts, then researchers can be relatively confident in the application of the task in the new setting. Given that MTurk workers tend to be more demographically diverse than college samples (Buhrmester et al., 2011), MTurk studies might even demonstrate better psychometric qualities than in-lab studies on college students.

The purpose of Issue 3 is to demonstrate the comparison of psychometric properties between the AX-CPT as administered in a laboratory setting and on the MTurk platform. To our knowledge, there is only one published study using the AX-CPT on MTurk, and this study used Dot Pattern Expectancy task. Like Crump et al. (2013), this study of the Dot Pattern Expectancy task did not report any psychometric measures or direct comparisons with a laboratory version (Otto et al., 2015).

### Methods

#### AX-CPT Datasets

The in-lab dataset used in this section comes from Experiment 2 in Gonthier et al. (2016). This is the same experiment detailed in Issue 2 above. While the Savoy dataset above used the standard condition of the AX-CPT, the in-lab dataset employed here in Issue 3 used a version of the AX-CPT that included no-go trials. The same 93 participants from Issue 2 completed this condition (the experiment was based on a within-subjects design). Going forward, this dataset will simply be referred to as the “in-lab” dataset. The task had 124 total trials: 40 AX, 10 AY, 10 BX, 40 AY, 12 NGA (no-go trials beginning with an “A” cue), and 12 NGB (no-go trials beginning with a “B” cue). Note that no-go trials will not be included in the assessment of reliability and variability in order to maintain consistency with the other datasets used in this manuscript, resulting in 100 total trials for the in-lab study.

The goal of the MTurk (previously unpublished) study was to assess the feasibility of administering the AX-CPT in an online manner. Sixty-five participants completed the task at two different time points; only the first session is examined here in order to equate it to the in-lab study. Participants found the experiment while browsing the MTurk platform for HITs. The description of the HIT contained an informed consent information sheet. Participants could then review the information and confirm their understanding and consent to the procedures by clicking the link at the bottom of the information sheet, effectively accepting the HIT, which then redirected to an external website hosted by the experimenters. The Washington University in St. Louis institutional review board approved this protocol. The AX-CPT task also included no-go trials, and had trial type proportions similar to the in-lab study: 72 AX, 18 AY, 18 BX, 72 BY, 18 NGA, and 18 NGB (216 total trials). Trials were removed in order to equate the total number of trials between the in-lab and MTurk tasks. This was done by keeping the first 40 AX and BY trials and the first 10 AY and BX trials, and removing the rest. Therefore, both datasets used in the present analyses contained 100 total trials. Of note, the proportion of females in each sample, as well as the mean and range of ages are different between the two datasets (**Table 5**).

**Table 5:** Descriptive statistics of AX-CPT accuracy and internal consistency reliability estimates: Issue 3.

Group	N subjects (% female)	Mean age (range)	Trial type	Mean accuracy	Variance × 10^-4^	Min	Max	α
In-lab	93 (78.5%)	20.18 (17–25)	AX	0.94	52.66	0.57	1.00	0.74
			AY	0.92	82.89	0.70	1.00	0.15
			BX	0.80	250.98	0.30	1.00	0.39
			BY	0.99	4.56	0.90	1.00	0.29
MTurk	65 (47.7%)	33.90 (21–53)	AX	0.93	84.79	0.45	1.00	0.82
			AY	0.93	92.21	0.60	1.00	0.29
			BX	0.83	200.87	0.40	1.00	0.35
			BY	0.98	10.05	0.82	1.00	0.62


#### Analyses

As in Issue 2, Brown–Forsythe tests were used to compare observed variances, internal consistency reliability was estimated via Cronbach’s α, and comparisons of α values was performed via Feldt tests.

### Results

**FIGURE 3:**
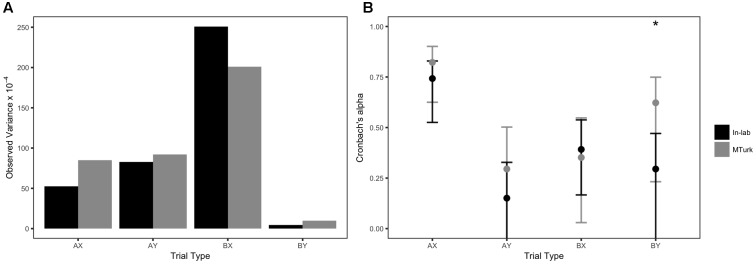
Observed variances and internal consistency reliability estimates of AX-CPT accuracy: Issue 3. Error bars represent 95% confidence intervals. Asterisk (^∗^) indicates significant Brown–Forsythe tests or significant Feldt tests at *p* < 0.05.

Descriptive statistics revealed that AX-CPT performance was very similar for both datasets, despite the MTurk study tapping a more diverse population and being administered online (**Table 5**). All Brown–Forsythe tests were non-significant for differences in observed variances between the two datasets (**Figure 3A**). Please see Supplementary Table S1c for skew and kurtosis values.

The MTurk dataset had a significantly higher Cronbach’s α than the in-lab study on BY trials [α = 0.62 and α = 0.29, respectively; χ^2^(1) = 1.87, *p* = 0.006], although neither coefficient would be considered “acceptable” by traditional psychometric standards. No other trial types exhibited any significant differences in reliability; however, it is worth noting that the MTurk dataset had numerically higher alphas than the in-lab study on AX, AY, and BY trial types (**Figure 3B**).

### Discussion

Overall, the comparison of in-lab and MTurk AX-CPT paradigms showed that the tasks exhibited similar psychometric properties. Data collected online in the MTurk sample even demonstrated significantly higher reliability for BY trials than data collected in-lab. This difference in reliability can again be interpreted in the context of a difference of variability: average performance was slightly lower for the MTurk sample, and most importantly, observed variance was 2.20 times larger for the MTurk sample than for the in-lab sample, although the difference did not reach significance. This may be partly attributable to differences in composition of the samples: as detailed in **Table 5**, the MTurk sample had a larger age range and more gender diversity. Generally speaking, MTurk samples have been shown to be more diverse (Buhrmester et al., 2011), and researchers often use MTurk and other online platforms specifically to reach a broader audience. The present results suggest that this difference of sample composition can also influence psychometric properties of the tasks.

Taken in the broader context of classic cognitive findings being replicated on MTurk (Germine et al., 2012; Crump et al., 2013), and evidence that MTurk workers can be more attentive (Hauser and Schwarz, 2015) than their in-lab counterparts, the lack of differences in psychometric characteristics of the AX-CPT observed here supports the notion that Internet studies can yield cognitive performance measurements that are as precise as in-lab studies. Though one could simply compare standard descriptive statistics, formal evaluation of psychometric properties ensures that precision of the measures is satisfying when administering a task in a new setting. We recommend doing so early on in order to (a) prevent scenarios in which surprising or misleading results are obtained due to differences in the task’s psychometric qualities (see Issues 1 and 2), and (b) contribute to ongoing replication efforts in the field of psychology (Open Science Collaboration, 2015).

## Issue 4: Psychometric Characteristics Can Help Optimize Study Design

Researchers are constantly tasked with making difficult study design decisions for both practical and theoretical reasons. The goal of Issue 4 is to demonstrate how careful examination of psychometric properties can aid scientists in making study design decisions that will provide maximal benefit for individual differences projects. To this end, we focus on a common problem faced by researchers designing studies: deciding on the number of trials to include in a task session.

Task length is always of concern in cognitive psychology, and is also one of the primary factors influencing psychometric properties. The classic Spearman–Brown Prophecy demonstrates how increasing the number of trials directly leads to an increase in reliability (Brown, 1910; Spearman, 1910). Conversely, scientists strive to collect psychometrically sound data while minimizing the time burden on participants. This has become especially important when combining multiple assessments into a single cognitive battery, as is becoming more frequent in the current “big data” era, and when administering tasks in out-of-the-lab environments (such as MTurk). Issue 4 explores how examining the psychometric properties of a task as a function of increasing the number of trials may help researchers better position themselves for capturing individual differences relationships. Here we compare the psychometric properties of the same MTurk dataset detailed in Issue 3, carved up into three separate datasets based on within-session blocks that include differing numbers of trials.

### Methods

#### AX-CPT Datasets

The same MTurk (unpublished) dataset used in Issue 3 is assessed here, this time using data from both sessions (test and retest). The mean test–retest interval was 2.34 days (±2.35 days, range of 14 h to 8 days). Each session consisted of three blocks of trials with equal numbers of trials per block: 24 AX, 6 AY, 6 BX, 24 BY, 6 NGA, and 6 NGB. As in Issue 3, no-go trials are not examined in the present study to maintain consistency. Thus, participants completed 60 trials of interest per block, and since each session consisted of three blocks, the total number of trials per session was 180 (excluding the 36 no-go trials).

In order to examine the effects of task length on psychometric properties of the AX-CPT, we assessed variability and reliability additively. The first dataset, described here as “Block 1,” included trials from only the first block (for both sessions). In other words, analyses of Block 1 included a total of 60 trials per session. The second dataset, or “Blocks 1 & 2,” included trials from only the first two blocks (1 and 2, for both sessions), for a total of 120 trials per session. The third dataset, “Blocks 1 & 2 & 3,” included the full dataset of 180 total trials per session.

#### Analyses

Differences in observed variances were again examined via Brown–Forsythe tests. We also obtained Cronbach’s α estimates for internal consistency within each session. Test–retest reliability was computed with the ICC to preserve comparability with Issue 1. As before, we considered non-overlapping 95% confidence intervals to be indicative of significant differences between two ICC values. Though we report both internal consistency reliability and test–retest reliability estimates, we focused on test–retest reliability in order to take advantage of the two sessions of data collection.

### Results

**FIGURE 4:**
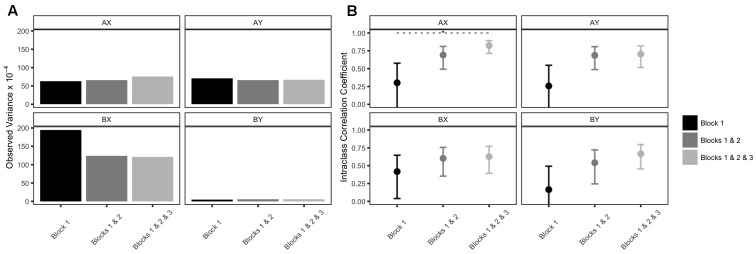
Observed variances and test–retest reliability estimates of AX-CPT accuracy: Issue 4. Error bars represent 95% confidence intervals. Asterisks (^∗^) indicate significant Brown–Forsythe tests at *p* < 0.05, or non-overlapping 95% confidence intervals.

Descriptive statistics are reported in **Table 6** (refer back to **Table 5** for the demographic information from this sample). Please see Supplementary Table S1d for skew and kurtosis values. AX-CPT accuracy remained quite stable across the three blocks of trials. Interestingly, increasing the number of trials did not impact observed variance substantially—no Brown–Forsythe tests revealed significant differences (**Figure 4A**).

**Table 6:** Descriptive statistics of AX-CPT accuracy: Issue 4.

Trial type	Blocks included	Trials per block	Mean accuracy	Variance × 10^-4^	Min	Max
AX	Block 1	24	0.94	63.32	0.52	1.00
	Blocks 1 & 2	48	0.92	66.40	0.67	1.00
	Blocks 1 & 2 & 3	72	0.92	76.03	0.58	1.00
AY	Block 1	6	0.93	70.51	0.67	1.00
	Blocks 1 & 2	12	0.93	65.52	0.62	1.00
	Blocks 1 & 2 & 3	18	0.92	66.80	0.58	1.00
BX	Block 1	6	0.85	194.38	0.50	1.00
	Blocks 1 & 2	12	0.86	124.26	0.58	1.00
	Blocks 1 & 2 & 3	18	0.86	121.41	0.56	1.00
BY	Block 1	24	0.99	4.84	0.90	1.00
	Blocks 1 & 2	48	0.98	5.94	0.84	1.00
	Blocks 1 & 2 & 3	72	0.98	6.07	0.83	1.00


**Table 7** contains all reliability estimates, and the **Figure 4B** plots test–retest ICCs. All reliability estimates—no matter how they were estimated—increased as a function of the number of trials, a clear demonstration of the Spearman–Brown Prophecy (Brown, 1910; Spearman, 1910). Internal consistency estimates were quite low for AY and BX trials, especially in the first session (T1; **Table 7**). ICCs were significantly different between Block 1 and Blocks 1 & 2 & 3 for AX trials (**Figure 4A**); all other 95% confidence intervals overlapped. The largest gains in ICCs were observed between the Block 1 and Blocks 1 & 2 datasets. That is, the difference in ICCs between Block 1 and Blocks 1 & 2 was larger than the difference in ICCs between Blocks 1 & 2 and Blocks 1 & 2 & 3 (**Figure 4B**). Overall, the same pattern emerged for internal consistency estimates, although some gains were still observed between Blocks 1 & 2 and Blocks 1 & 2 & 3.

**Table 7:** Internal consistency and test–retest reliability estimates: Issue 4.

Trial type	Blocks included	T1 α	T2 α	ICC
AX	Block 1	0.88	0.61	**0.30**
	Blocks 1 & 2	0.83	0.86	**0.69**
	Blocks 1 & 2 & 3	0.88	0.91	**0.83**
AY	Block 1	0.20	0.20	**0.26**
	Blocks 1 & 2	0.40	0.33	**0.69**
	Blocks 1 & 2 & 3	0.44	0.66	**0.70**
BX	Block 1	0.19	0.49	**0.42**
	Blocks 1 & 2	0.39	0.51	**0.60**
	Blocks 1 & 2 & 3	0.56	0.68	**0.63**
BY	Block 1	0.52	0.25	**0.16**
	Blocks 1 & 2	0.74	0.45	**0.54**
	Blocks 1 & 2 & 3	0.79	0.56	**0.67**


### Discussion

Studies are often limited by time. The goal of Issue 4 was to demonstrate that researchers might be motivated to make different study design decisions based on psychometric information. As predicted by the Spearman–Brown formula (Brown, 1910; Spearman, 1910), reliability increased as a function of the number of trials. Gains in reliability were especially important from one block (60 total trials) to two blocks (120 total trials); there was minimal added benefit from including the extra block (**Figure 4B**). Given these data, a researcher may decide that only two blocks per session are sufficient, and choose not to administer a full three blocks per session (180 total trials). The full picture is a little more complex, however, as certain reliability indices only approached a satisfying range with the addition of the third block (e.g., test–retest reliability for BY trials). This finding implies that the appropriate number of trials when designing the task can vary, depending on which performance indices and which trial types are of interest.

This type of preliminary analysis allows researchers to minimize practical testing constraints, as well as the participant’s burden, without sacrificing psychometric quality. To be fair, these data are presented in a relatively coarse manner, and there are a number of ways one could have gone about examining the question of task length (e.g., iteratively re-computing after each sequential trial). Future studies could explore a more systematic approach to addressing questions of task length, such as meticulously examining the change in variability and reliability as a function of iteratively adding a few trials (rather than adding a whole block, as we did here).

Although Issue 4 specifically highlighted task length, evaluation of psychometric characteristics of a task can be used for other study design questions. For example, many studies based on the AX-CPT have used measures of performance other than accuracy, including RTs, composite scores, and signal detection theory indices (e.g., *d*′-context). *A priori* evaluation of the psychometric characteristics of these various measures could help researchers select the best index to use in individual differences analyses. Though we report the same analyses from all four Issues using *d*′-context instead in Supplementary Material, future studies may want to more rigorously compare and contrast the variability and reliability of each performance measure.

## General Discussion

The present study carefully examined various datasets of the AX-CPT in order to illustrate common issues that researchers may encounter when preparing to use a cognitive task for an individual differences study. Issues 1 and 2 demonstrated how differential correlations could potentially reflect an artifact due to the task exhibiting different psychometric characteristics between populations (Issue 1) or even between samples within the same population (Issue 2). Such problems may even directly contribute to failure of a study to replicate a hypothesized effect (Issue 2). Issue 3 demonstrated how evaluating the psychometric properties of a task can help researchers decide whether a task is appropriate for use in new settings or environments, such as going from an in-lab to online administration technique. Finally, Issue 4 illustrated that evaluation of psychometric characteristics can help researchers make important study design issues, such as those pertaining to overall task length.

The findings presented here convey the importance of scrutinizing the psychometric properties of cognitive tasks, especially those intended for use in individual differences studies. Reliability is not always reported or even examined by researchers, and the present results also demonstrate why variability and reliability should be considered and reported in the context of one another. Future research efforts related to individual differences in cognition could benefit from incorporating psychometric techniques. This may hopefully lead to improvements in the design and evaluation of cognitive tasks for the purposes of individual differences research.

It could be the case that researchers working with cognitive tasks in experimental psychology are less used to examining the psychometric qualities of their measures than researchers in other fields. At this point, it may be worthwhile to recall here that psychometric theory is not inherently less well-aligned with cognitive psychology than, say, personality research. On the contrary, CTT and concepts such as reliability were initially designed for experimental tasks to be used in cognitive research (e.g., Spearman, 1904). For this reason, the same standards apply to measures collected in experimental cognitive tasks than to any other measurement in psychology. In fact, what constitutes acceptable psychometric properties depends on the intended purpose of the task, not on the type of construct it is supposed to measure. Nunnally (1978) famously defined a reliability coefficient of 0.70 as “modest reliability,” acceptable “in the early stages of research”; reliability of 0.80 as adequate for basic research; and reliability of 0.90 as “the minimum that should be tolerated” in “those applied settings where important decisions are made with respect to specific test scores.” The present case study shows that several indices commonly used with the AX-CPT routinely fail to meet these standards by a large margin (e.g., α = 0.15 for AY trials in the in-lab dataset in Issue 3), which warrants systematic inspection by researchers.

### Implications for AX-CPT Research

While the four issues presented here illustrate the need for detailed analysis of task properties, little attention has been devoted to psychometric validation of the AX-CPT in the literature. The task was initially developed for use with clinical samples (Rosvold et al., 1956; Servan-Schreiber et al., 1996), in a series of studies that focused on its ability to discriminate between lesioned and control participants; formal psychometric validation was not completed in these initial endeavors. Later studies produced numerous versions of the paradigm (e.g., Redick, 2014; Richmond et al., 2015), adapted the task for other populations (such as children and older adults; Braver et al., 2005; Chatham et al., 2009), and developed multiple indices of performance (e.g., Gonthier et al., 2016); psychometric properties were not considered in detail in these subsequent publications either. Although reliability of the AX-CPT has been explored in considerable detail in task variants optimized for use in schizophrenia samples (Henderson et al., 2011; Strauss et al., 2014), reliability has remained relatively under-explored in healthy adult samples.

As for the present case study, it was limited by its retrospective evaluation; AX-CPT datasets were already collected for different research purposes, and thus had variable task properties such as overall length and trial frequencies. We tried to control for this by matching studies appropriately, such as only comparing datasets with no-go trials to other datasets with no-go trials, and ensuring trial type frequencies were similar (as in Issue 2) or equal (as in Issue 3). Yet this limitation, and the very existence of important variations between versions of the task across studies, highlights the need for more dedicated efforts at psychometric validation in future research.

The problem illustrated in Issue 2 is especially relevant to AX-CPT research, as it questions the replicability of results obtained with the task. It seems very unlikely that slight variations in task structure can explain the differences in psychometric qualities of the AX-CPT when administered to two samples of college students (Savoy vs. Temple). These datasets had nearly identical trial proportions (with slightly fewer overall trials in the Savoy sample) and were conducted in identical contexts (in-lab). As mentioned in the Issue 2 discussion, the Savoy participants were very close to accuracy ceiling levels, though it is still unclear why Savoy participants were at ceiling and Temple participants were not. It is possible that demographic information not normally collected and/or analyzed in studies with samples mainly comprised of college students (i.e., socioeconomic status, major, parental education, etc.) is responsible for this discrepancy; or perhaps differences in the ability level or motivation of the two samples contributed. In any case, these results indicate that subtle differences can exist between samples that appear, on the surface, to be similar, and can create unexpected ceiling effects. This point highlights the need for examination of psychometric properties in each specific sample where the task is used. Given these findings, it might also be worthwhile for future studies to explore more in-depth if and how demographic information or general ability and motivation levels relate to AX-CPT performance.

### The AX-CPT as a Case Study

The AX-CPT was chosen as a case study because it is representative of a flavor of cognitive tasks—especially cognitive control tasks—that may be especially vulnerable to psychometric issues. The cognitive tasks most at risk are those that elicit limited variability, usually due to ceiling effects, and those in which scores are based on infrequent trial types. The former category includes tasks designed for clinical populations, which are then often used with non-clinical participants; this is notably the case for certain tasks within the domain of executive function (see Miyake et al., 2000). The latter category comprises many paradigms developed in the context of cognitive control research. The list includes tasks with unbalanced proportions of congruent and incongruent trials (such as variants of the Stroop task: Logan and Zbrodoff, 1979; and the Flanker task: Lehle and Hübner, 2008), and go/no-go tasks with very few no-go trials (e.g., the Sustained Attention to Response Task; Robertson et al., 1997). Therefore, the conclusions of the present case study can easily generalize to a wide range of commonly used paradigms, thus emphasizing the need to carefully examine psychometric properties of these types of tasks.

Given that these types of tasks are particularly at risk, how can researchers go about optimizing the psychometric qualities of their paradigms? Research using the AX-CPT suggests at least three ways of circumventing issues of variability and reliability in cognitive control tasks. A first possibility is to increase the number of trials, as illustrated in Issue 4. Increasing test length directly reduces the proportion of error variance and has always constituted a key solution to increase reliability (e.g., Nunnally, 1967). However, we urge researchers to exercise caution when increasing the task length, since reliability will only increase if the measurement error is random. If the task becomes too long and participants become overly bored or tired, then the measurement error may systematically increase, ultimately decreasing reliability. Consequently, increasing the length of the task as a means to increase reliability is a viable option, to the extent that other sources of measurement error (e.g., participant boredom or fatigue) remain minimized by such manipulations. On the other hand, increasing the number of trials can be particularly impractical in the context of cognitive control tasks with infrequent trial types, because it can disproportionately inflate the duration of the task. A second possibility is to extend participant selection beyond college students and to recruit a diverse sample (e.g., from the community). Issue 3 provides an example of how this can be achieved through online data collection. As demonstrated, increasing variability in the sample increases reliability of the measures, all else being equal. This solution has the advantage of being relatively straightforward, while also helping researchers generalize their findings. A third possibility is to use experimental manipulations of the task to increase variability of the scores. In the context of the AX-CPT, for instance, experimental manipulations have been used to push non-clinical young participants off ceiling, such as including no-go trials in the task to reduce levels of proactive control and decrease performance on BX trials (see Gonthier et al., 2016). By increasing variability of participant scores, this type of manipulation could contribute to enhancing reliability.

### Classical Test Theory and Beyond

At least two aspects of the AX-CPT paradigm can be discussed in ways that depart from CTT. First, CTT considers all trials within a given task as independent events; all trials of a given type are supposed to be identical and interchangeable. This is likely not accurate in the case of the AX-CPT and similar tasks: it is very possible that certain sequences of items have specific features that alter their psychometric properties. Such sequences of items may be more indicative of a person’s true performance than other items, or even reflect different abilities altogether. For example, AY or BX trials following strings of target AX trials (AX-AX-AX-AX-**AY**) may provide different information than AY or BX trials following strings of BY trials (BY-BY-BY-BY-**AY**). Although the two AY trials in the previous example are treated as interchangeable in CTT, response to the AY trial in the first sequence is likely going to be impacted by the strong prepotency effect caused by the four previous AX trials, whereas the AY trial in the second sequence will be exposed to no such prepotency effect (the opposite may actually be true). For this reason, the first sequence might be better at discriminating participants in the high ability range, or it might even be sensitive to different abilities altogether, such as response inhibition. Although some researchers have expressed concerns regarding trial order sequences in the AX-CPT (Chatham et al., 2009; Henderson et al., 2011), there has been no systematic study to determine whether high conflict trials (AY/BX) yield meaningfully different performance if placed in different parts of a string of trials. Yet such sequence effects have been shown on multiple occasions in cognitive control tasks (Gratton et al., 1992; Egner, 2007), and their impact may be amplified by the small number of conceptually interesting AY and BX trials.

The effects of randomized trial type presentation in creating variable sequences of stimuli are effectively removed when performance is averaged across individuals, as is usually the case in experimental designs. However, presentation of randomized stimuli sequences creates problems for individual differences questions, since each subject is effectively completing a different task with a different underlying task structure. Presenting stimuli in a fixed sequence, rather than a randomized order, can help reduce this confound. For example, Henderson et al. (2011) opted to pseudorandomly create a fixed sequence of trials that was then administered to all participants. This is an interesting first step, although whether a fixed trial order is especially useful for improving the psychometric characteristics of the task remains to be determined. Another possible approach to sequences of stimuli is item response theory (IRT), a prominent psychometric framework specifically used in measurement construction to precisely estimate difficulty and discrimination of specific items—or in the present case, sequences of items. IRT could be used to create an adaptive version of the AX-CPT, administering the most discriminating sequences of items for a given participant. For example, a subject with high cognitive control performance in the task could be confronted with progressively more difficult sequences of trials, ultimately resulting in more efficient categorizations of individuals. Though IRT methods have been effectively used in educational assessments, it is not straightforward to adapt them for use in standard cognitive tasks. Operationalizing this approach while preserving the relative proportions of different trial types may be challenging, but it could prove a worthwhile endeavor in future studies.

A second way that the AX-CPT departs from CTT concerns reliability and the nature of measurement error. We discussed reliability in the context of the CTT framework, which constitutes the most common approach in the cognitive literature. However, there are newer formulations of psychometric theory that may be relevant for questions relating to individual differences in cognition. As briefly described in the introduction, the well-known CTT equation decomposes observed variance into true score variance and error variance. Another framework, Generalizability Theory (G-Theory), allows for a more flexible approach of measurement error (Cronbach et al., 1963, 1972). G-theory posits that error variation can occur due to a number of sources or *facets*, such as time, nature of the test items, and nature of the task. Contrary to CTT, which summarizes random variance in a single error term, G-theory aims to quantify the amount of random variance attributable to the different facets. For instance, a typical generalizability study could compare measures obtained with different versions of the task and at different time points. This information can then be used to make decisions regarding the design of the task, in order to improve upon reliability under researcher-defined conditions and ultimately increase true score variance. Those interested in pursuing individual differences questions may want to use G-theory in order to gain a better understanding of the multiple sources of error. This could be especially useful in the context of AX-CPT research, where multiple versions of the task coexist and where test–retest protocols are used on a regular basis.

## Author Contributions

SC and CG contributed to the conception and study design, data collection, data analysis and interpretation, drafting the article, critical revision of the article, and final approval of the version to be published. DB and TB contributed to the conception and study design, data analysis and interpretation, drafting the article, critical revision of the article, and final approval of the version to be published.

## Conflict of Interest Statement

The authors declare that the research was conducted in the absence of any commercial or financial relationships that could be construed as a potential conflict of interest.
