# Supplementary material for: The Role of Psychometrics in Individual Differences Research in Cognition: A Case Study of the AX-CPT
Source: Front Psychol. 2017 Sep 4;8:1482. doi: 10.3389/fpsyg.2017.01482 (PMC5591582; doi:10.3389/fpsyg.2017.01482)
Supplement: Supplementary file 1 [file Data_Sheet_1.DOCX]

**The Role of Psychometrics in Individual Differences Research in Cognition: A Case Study of the AX-CPT – Supplement Materials**

All reaction time data reflect correct trials only. The three derived measures include *d’*-context, the Proactive Behavioral Index for error rates (PBI ER), and the Proactive Behavioral Index for reaction times (PBI RT). The *d’* index comes from signal detection theory (Stanislaw & Todorov, 1999), and is used to indicate how well a subject utilizes cue information (“context”) for X-probe responses (i.e., AX vs. BX trials). The *d’*-context was calculated using the formula Z(AX hits) – Z(BX false alarms), where Z represents the z-transform of the value. The PBI was developed by Braver and colleagues (2009) in order to assess the degree of proactive or reactive control within an individual, and is calculated by (AY – BX) / (AY + BX). A positive PBI value indicates that the subject engages in proactive control, as marked by higher AY interference, whereas a negative PBI value indicates that the subject engages in reactive control, as marked by higher BX interference (Braver, Paxton, Locke, & Barch, 2009; Gonthier, Macnamara, Chow, Conway, & Braver, 2016). The PBI was obtained using both error rates and reaction times (PBI ER and PBI RT, respectively). Note that for *d’*-context and PBI ER, we applied a log-linear correction such that error rate = (number of errors + 0.5) / (number of trials + 1) (Hautus, 1995). Due to operational constraints, internal consistency reliabilities presented in this Supplement were obtained via boostrapped split half correlations (1000 iterations; including Spearman-Brown correction), as opposed to Cronbach’s *α*.

References:

Braver, T. S., Paxton, J. L., Locke, H. S., & Barch, D. M. (2009). Flexible neural mechanisms of cognitive control within human prefrontal cortex. *Proceedings of the National Academy of Sciences*, *106*(18), 7351–7356. http://doi.org/10.1073/pnas.0808187106

Gonthier, C., Macnamara, B. N., Chow, M., Conway, A. R. A., & Braver, T. S. (2016). Inducing Proactive Control Shifts in the AX-CPT. *Frontiers in Psychology*, *7*, 1822. http://doi.org/10.3389/fpsyg.2016.01822

Hautus, M. J. (1995). Corrections for extreme proportions and their biasing effects on estimated values ofd′. *Behavior Research Methods, Instruments, & Computers*, *27*(1), 46–51. http://doi.org/10.3758/BF03203619

Stanislaw, H., & Todorov, N. (1999). Calculation of signal detection theory measures. *Behavior Research Methods, Instruments, & Computers*, *31*(1), 137–149. http://doi.org/10.3758/BF03207704

**Supplement 1 – Skew and Kurtosis Tables**

For each of the four issues described in the main text, a skew and kurtosis table is provided below. Values corresponding to accuracy are presented in bold, as these correspond to the descriptive tables presented in the main text.

Table S1a. *Skew and Kurtosis for all metrics: Issue 1*

| Group | Metric | Trial Type | Skew | Kurtosis |
| --- | --- | --- | --- | --- |
| CTRL | **Accuracy** | **AX** | **-1.75** | **3.25** |
|  |  | **AY** | **-2.07** | **4.72** |
|  |  | **BX** | **-2.00** | **4.93** |
|  |  | **BY** | **-3.79** | **17.23** |
|  | Reaction Time | AX | .45 | -.13 |
|  |  | AY | .51 | -.15 |
|  |  | BX | .76 | .64 |
|  |  | BY | .40 | -.32 |
|  | Derived Measures | *d*’-context | -.91 | .31 |
|  |  | PBI ER | -.03 | -.08 |
|  |  | PBI RT | -.35 | -.26 |
| SCZ | **Accuracy** | **AX** | **-2.55** | **8.27** |
|  |  | **AY** | **-1.66** | **2.36** |
|  |  | **BX** | **-1.63** | **3.12** |
|  |  | **BY** | **-4.73** | **30.57** |
|  | Reaction Time | AX | .93 | 1.90 |
|  |  | AY | .43 | -.27 |
|  |  | BX | 1.12 | 1.56 |
|  |  | BY | .43 | .06 |
|  | Derived Measures | *d*’-context | -.44 | -.29 |
|  |  | PBI ER | -.24 | -.93 |
|  |  | PBI RT | .07 | -.46 |

Table S1b. *Skew and Kurtosis for all metrics: Issue 2*

| Group | Metric | Trial Type | Skew | Kurtosis |
| --- | --- | --- | --- | --- |
| Savoy | **Accuracy** | **AX** | **-1.97** | **4.97** |
|  |  | **AY** | **-1.11** | **.59** |
|  |  | **BX** | **-1.54** | **1.97** |
|  |  | **BY** | **-1.81** | **2.75** |
|  | Reaction Time | AX | .51 | -.44 |
|  |  | AY | .23 | -.58 |
|  |  | BX | .85 | .88 |
|  |  | BY | .72 | 1.39 |
|  | Derived Measures | *d*’-context | -.70 | .43 |
|  |  | PBI ER | -.17 | -1.06 |
|  |  | PBI RT | -.46 | .83 |
| Temple | **Accuracy** | **AX** | **-1.77** | **3.20** |
|  |  | **AY** | **-.76** | **-.01** |
|  |  | **BX** | **-2.04** | **4.25** |
|  |  | **BY** | **-3.58** | **17.48** |
|  | Reaction Time | AX | 1.77 | 6.27 |
|  |  | AY | .43 | -.48 |
|  |  | BX | .98 | .94 |
|  |  | BY | .44 | .20 |
|  | Derived Measures | *d*’-context | -.72 | -.19 |
|  |  | PBI ER | -.17 | -1.04 |
|  |  | PBI RT | -.15 | -.44 |

Table S1c. *Skew and Kurtosis for all metrics: Issue 3*

| Group | Metric | Trial Type | Skew | Kurtosis |
| --- | --- | --- | --- | --- |
| In-lab | **Accuracy** | **AX** | **-2.34** | **7.03** |
|  |  | **AY** | **-.97** | **-.02** |
|  |  | **BX** | **-.69** | **.00** |
|  |  | **BY** | **-1.99** | **4.40** |
|  | Reaction Time | AX | .87 | .60 |
|  |  | AY | .31 | .07 |
|  |  | BX | .97 | 1.00 |
|  |  | BY | .83 | 1.78 |
|  | Derived Measures | *d*’-context | -.25 | .24 |
|  |  | PBI ER | .69 | -.42 |
|  |  | PBI RT | -.41 | -.02 |
| MTurk | **Accuracy** | **AX** | **-2.54** | **9.28** |
|  |  | **AY** | **-1.40** | **1.44** |
|  |  | **BX** | **-.86** | **.29** |
|  |  | **BY** | **-2.80** | **9.40** |
|  | Reaction Time | AX | 1.62 | 3.02 |
|  |  | AY | 1.87 | 3.93 |
|  |  | BX | 1.14 | 2.02 |
|  |  | BY | 2.41 | 10.09 |
|  | Derived Measures | *d*’-context | -.16 | -.86 |
|  |  | PBI ER | .56 | -.73 |
|  |  | PBI RT | .28 | .49 |

Table S1d. *Skew and Kurtosis for all metrics: Issue 4*

| Metric | Trial Type | Blocks Included | Skew | Kurtosis |
| --- | --- | --- | --- | --- |
| **Accuracy** | **AX** | **Block 1** | **-2.64** | **9.84** |
|  |  | **Blocks 1 & 2** | **-1.48** | **1.56** |
|  |  | **Blocks 1 & 2 & 3** | **-1.87** | **3.37** |
|  | **AY** | **Block 1** | **-1.33** | **1.39** |
|  |  | **Blocks 1 & 2** | **-1.50** | **2.59** |
|  |  | **Blocks 1 & 2 & 3** | **-1.99** | **4.82** |
|  | **BX** | **Block 1** | **-.68** | **-.62** |
|  |  | **Blocks 1 & 2** | **-.65** | **-.55** |
|  |  | **Blocks 1 & 2 & 3** | **-.68** | **-.32** |
|  | **BY** | **Block 1** | **-1.97** | **3.78** |
|  |  | **Blocks 1 & 2** | **-3.16** | **14.56** |
|  |  | **Blocks 1 & 2 & 3** | **-3.57** | **18.44** |
| Reaction Time | AX | Block 1 | 1.86 | 5.65 |
|  |  | Blocks 1 & 2 | 1.88 | 5.31 |
|  |  | Blocks 1 & 2 & 3 | 1.77 | 4.66 |
|  | AY | Block 1 | 1.92 | 4.94 |
|  |  | Blocks 1 & 2 | 2.64 | 9.46 |
|  |  | Blocks 1 & 2 & 3 | 2.87 | 11.32 |
|  | BX | Block 1 | .94 | .46 |
|  |  | Blocks 1 & 2 | .94 | 1.61 |
|  |  | Blocks 1 & 2 & 3 | .72 | .55 |
|  | BY | Block 1 | 2.58 | 11.04 |
|  |  | Blocks 1 & 2 | 2.68 | 12.06 |
|  |  | Blocks 1 & 2 & 3 | 1.98 | 7.25 |
| Derived Measures | *d*’-context | Block 1 | -.40 | -.46 |
|  |  | Blocks 1 & 2 | -.25 | -.89 |
|  |  | Blocks 1 & 2 & 3 | -.24 | -.83 |
|  | PBI ER | Block 1 | .45 | -.79 |
|  |  | Blocks 1 & 2 | .47 | -.65 |
|  |  | Blocks 1 & 2 & 3 | .43 | -.70 |
|  | PBI RT | Block 1 | .50 | .97 |
|  |  | Blocks 1 & 2 | 1.43 | 3.91 |
|  |  | Blocks 1 & 2 & 3 | 1.42 | 4.26 |

**Supplement 2 –** **Issue 1: Psychometric properties of a measure can complicate between-populations findings**

Table S2a. *Descriptive statistics of AX-CPT reaction time, Proactive Behavioral Shift with error rates, and Proactive Behavioral Shift with reaction time: Issue 1*

| Group | Metric | Trial Type | Mean | Variance | Min | Max |
| --- | --- | --- | --- | --- | --- | --- |
| CTRL  (n = 119) | Raw RT | AX | 442.42 | 3679.27 | 316.80 | 629.30 |
|  |  | AY | 558.17 | 3935.77 | 426.36 | 729.30 |
|  |  | BX | 426.49 | 9532.20 | 225.12 | 750.03 |
|  |  | BY | 430.39 | 6969.23 | 260.12 | 649.41 |
|  | Derived Measures | *d*'-context | 3.42 | .56 | 1.09 | 4.48 |
|  |  | PBI ER | -.09 | .09 | -.81 | .78 |
|  |  | PBI RT | .14 | .01 | -.09 | .34 |
| SCZ  (n = 92) | Raw RT | AX | 477.90 | 7175.17 | 316.98 | 823.85 |
|  |  | AY | 588.41 | 7497.00 | 413.75 | 797.00 |
|  |  | BX | 508.25 | 24181.58 | 263.56 | 1048.67 |
|  |  | BY | 486.67 | 11153.72 | 271.00 | 753.86 |
|  | Derived Measures | *d*'-context | 2.74 | .96 | .19 | 4.48 |
|  |  | PBI ER | -.08 | .13 | -.91 | .64 |
|  |  | PBI RT | .09 | .01 | -.16 | .36 |

Table S2b. *Internal consistency and test-retest reliability of AX-CPT reaction time, Proactive Behavioral Shift with error rates, and Proactive Behavioral Shift with reaction time: Issue 1*

| Group | Metric | Trial Type | T1  SHC | T2  SHC | T3  SHC | T1-T2-T3 ICC |
| --- | --- | --- | --- | --- | --- | --- |
| CTRL | Raw RT | AX | .97 | .98 | .98 | .88 |
|  |  | AY | .87 | .91 | .90 | .92 |
|  |  | BX | .90 | .90 | .85 | .86 |
|  |  | BY | .83 | .85 | .80 | .84 |
|  | Derived Measures | *d’*-context | .85 | .78 | .83 | .80 |
|  |  | PBI ER | .51 | .38 | .40 | .38 |
|  |  | PBI RT | .84 | .84 | .77 | .82 |
| SCZ | Raw RT | AX | .98 | .98 | .98 | .92 |
|  |  | AY | .91 | .91 | .91 | .91 |
|  |  | BX | .93 | .93 | .93 | .88 |
|  |  | BY | .87 | .87 | .86 | .90 |
|  | Derived Measures | *d’*-context | .86 | .89 | .90 | .86 |
|  |  | PBI ER | .49 | .44 | .61 | .72 |
|  |  | PBI RT | .84 | .86 | .84 | .83 |

T – time point, SHC – split half correlation, ICC – intraclass correlation coefficient, CTRL – control group, SCZ – schizophrenia group.

**Supplement 3 –** **Issue 2: Psychometric characteristics of a task can impact replication attempts**

Table S3a. *Descriptive statistics of AX-CPT reaction time, Proactive Behavioral Shift with error rates, and Proactive Behavioral Shift with reaction time: Issue 2*

| Group | Metric | Trial Type | Mean | Variance | Min | Max |
| --- | --- | --- | --- | --- | --- | --- |
| Savoy  (n = 93) | Raw RT | AX | 384.66 | 1964.21 | 296.38 | 490.31 |
|  |  | AY | 464.77 | 2896.61 | 351.70 | 595.80 |
|  |  | BX | 375.23 | 7837.50 | 193.20 | 677.80 |
|  |  | BY | 351.89 | 3574.93 | 235.18 | 592.67 |
|  | Derived Measures | *d*'-context | 3.11 | .37 | 1.13 | 3.94 |
|  |  | PBI ER | .11 | .20 | -.75 | .83 |
|  |  | PBI RT | .12 | .01 | -.15 | .35 |
| Temple  (n = 104) | Raw RT | AX | 478.64 | 5335.22 | 356.80 | 862.48 |
|  |  | AY | 554.11 | 3394.92 | 441.33 | 691.45 |
|  |  | BX | 481.33 | 11159.85 | 308.71 | 811.92 |
|  |  | BY | 439.60 | 3930.02 | 301.54 | 636.81 |
|  | Derived Measures | *d*'-context | 2.42 | .95 | -.26 | 4.22 |
|  |  | PBI ER | .00 | .23 | -.92 | .75 |
|  |  | PBI RT | .08 | .01 | -.15 | .26 |

Table S3b. *Internal consistency reliability of AX-CPT reaction time, Proactive Behavioral Shift with error rates, and Proactive Behavioral Shift with reaction time: Issue 2*

| Group | Metric | Trial Type | SHC |
| --- | --- | --- | --- |
| Savoy | Raw RT | AX | .91 |
|  |  | AY | .79 |
|  |  | BX | .79 |
|  |  | BY | .94 |
|  | Derived Measures | *d’*-context | .51 |
|  |  | PBI ER | .38 |
|  |  | PBI RT | .60 |
| Temple | Raw RT | AX | .94 |
|  |  | AY | .77 |
|  |  | BX | .81 |
|  |  | BY | .96 |
|  | Derived Measures | *d’*-context | .84 |
|  |  | PBI ER | .52 |
|  |  | PBI RT | .64 |

SHC – split half correlation

**Supplement 4 –** **Issue 3: Psychometric characteristics can help decide on a data collection method**

Table S4a. *Descriptive statistics of AX-CPT reaction time, Proactive Behavioral Shift with error rates, and Proactive Behavioral Shift with reaction time: Issue 3*

| Group | Metric | Trial Type | Mean | Variance | Min | Max |
| --- | --- | --- | --- | --- | --- | --- |
| In-lab  (n = 93) | Raw RT | AX | 434.50 | 3327.44 | 334.58 | 615.87 |
|  |  | AY | 541.92 | 3385.89 | 395.62 | 694.11 |
|  |  | BX | 511.50 | 11395.48 | 342.38 | 868.71 |
|  |  | BY | 442.66 | 2699.58 | 330.36 | 647.08 |
|  | Derived Measures | *d*'-context | 2.44 | .53 | .41 | 3.94 |
|  |  | PBI ER | -.29 | .18 | -.86 | .75 |
|  |  | PBI RT | .04 | .01 | -.22 | .26 |
| MTurk  (n = 65) | Raw RT | AX | 491.97 | 8616.71 | 384.14 | 827.13 |
|  |  | AY | 596.36 | 15038.98 | 432.11 | 1025.44 |
|  |  | BX | 585.73 | 19053.10 | 379.33 | 1102.67 |
|  |  | BY | 482.76 | 8016.84 | 341.33 | 958.95 |
|  | Derived Measures | *d*'-context | 2.54 | .67 | .81 | 3.94 |
|  |  | PBI ER | -.24 | .18 | -.83 | .75 |
|  |  | PBI RT | .01 | .01 | -.20 | .32 |

Table S4b. *Internal consistency reliability of AX-CPT reaction time, Proactive Behavioral Shift with error rates, and Proactive Behavioral Shift with reaction time: Issue 3*

| Group | Metric | Trial Type | SHC |
| --- | --- | --- | --- |
| In-lab | Raw RT | AX | .91 |
|  |  | AY | .68 |
|  |  | BX | .70 |
|  |  | BY | .92 |
|  | Derived Measures | *d’*-context | .55 |
|  |  | PBI ER | .34 |
|  |  | PBI RT | .51 |
| MTurk | Raw RT | AX | .94 |
|  |  | AY | .87 |
|  |  | BX | .55 |
|  |  | BY | .94 |
|  | Derived Measures | *d’*-context | .66 |
|  |  | PBI ER | .25 |
|  |  | PBI RT | .35 |

SHC – split half correlation

**Supplement 5 –** **Issue 4: Psychometric characteristics can help optimize study design**

Table S5a. *Descriptive statistics of AX-CPT reaction time, Proactive Behavioral Shift with error rates, and Proactive Behavioral Shift with reaction time: Issue 4*

| Metric | Trial Type | Blocks Included | Mean | Variance | Min | Max |
| --- | --- | --- | --- | --- | --- | --- |
| Raw RT | AX | Block 1 | 474.51 | 6414.99 | 372.10 | 839.51 |
|  |  | Blocks 1 & 2 | 477.65 | 7137.39 | 369.57 | 858.74 |
|  |  | Blocks 1 & 2 & 3 | 476.68 | 7205.40 | 357.17 | 842.03 |
|  | AY | Block 1 | 575.82 | 10951.22 | 439.55 | 1025.92 |
|  |  | Blocks 1 & 2 | 567.38 | 11161.55 | 441.73 | 1092.96 |
|  |  | Blocks 1 & 2 & 3 | 561.32 | 10951.07 | 427.31 | 1113.03 |
|  | BX | Block 1 | 561.77 | 14115.89 | 382.75 | 953.90 |
|  |  | Blocks 1 & 2 | 552.69 | 12357.70 | 344.00 | 975.47 |
|  |  | Blocks 1 & 2 & 3 | 537.24 | 11325.63 | 317.88 | 884.93 |
|  | BY | Block 1 | 471.80 | 6145.33 | 365.07 | 894.89 |
|  |  | Blocks 1 & 2 | 461.46 | 5892.12 | 359.17 | 885.02 |
|  |  | Blocks 1 & 2 & 3 | 453.59 | 4723.72 | 346.70 | 792.09 |
| Derived Measures | *d*'-context | Block 1 | 2.52 | .45 | .83 | 3.52 |
|  |  | Blocks 1 & 2 | 2.64 | .56 | .95 | 3.86 |
|  |  | Blocks 1 & 2 & 3 | 2.68 | .63 | .95 | 4.19 |
|  | PBI ER | Block 1 | -.17 | .12 | -.65 | .62 |
|  |  | Blocks 1 & 2 | -.19 | .14 | -.83 | .67 |
|  |  | Blocks 1 & 2 & 3 | -.20 | .16 | -.87 | .67 |
|  | PBI RT | Block 1 | .02 | .01 | -.16 | .32 |
|  |  | Blocks 1 & 2 | .02 | .01 | -.11 | .37 |
|  |  | Blocks 1 & 2 & 3 | .02 | .01 | -.12 | .38 |

Table S5b. *Internal consistency and test-retest reliability of AX-CPT reaction time, Proactive Behavioral Shift with error rates, and Proactive Behavioral Shift with reaction time: Issue 4*

| Metric | Trial Type | Blocks Included | T1 SHC | T2 SHC | T1-T2  ICC |
| --- | --- | --- | --- | --- | --- |
| Raw RT | AX | Block 1 | .91 | .94 | .79 |
|  |  | Blocks 1 & 2 | .95 | .96 | .83 |
|  |  | Blocks 1 & 2 & 3 | .97 | .97 | .86 |
|  | AY | Block 1 | .79 | .86 | .74 |
|  |  | Blocks 1 & 2 | .88 | .90 | .83 |
|  |  | Blocks 1 & 2 & 3 | .91 | .93 | .90 |
|  | BX | Block 1 | .42 | .60 | .72 |
|  |  | Blocks 1 & 2 | .62 | .75 | .75 |
|  |  | Blocks 1 & 2 & 3 | .75 | .83 | .82 |
|  | BY | Block 1 | .90 | .93 | .84 |
|  |  | Blocks 1 & 2 | .94 | .97 | .86 |
|  |  | Blocks 1 & 2 & 3 | .95 | .97 | .87 |
| Derived Measures | *d*'-context | Block 1 | .59 | .64 | .54 |
|  |  | Blocks 1 & 2 | .71 | .76 | .71 |
|  |  | Blocks 1 & 2 & 3 | .77 | .82 | .79 |
|  | PBI ER | Block 1 | .18 | .52 | .55 |
|  |  | Blocks 1 & 2 | .37 | .47 | .55 |
|  |  | Blocks 1 & 2 & 3 | .40 | .58 | .61 |
|  | PBI RT | Block 1 | .12 | .35 | .55 |
|  |  | Blocks 1 & 2 | .44 | .55 | .55 |
|  |  | Blocks 1 & 2 & 3 | .63 | .65 | .61 |

T – time point, SHC – split half correlation, ICC – intraclass correlation coefficient.
